# Editorial: Illuminating Carotenoid Synthesis and Plastid Transition in Plants

**DOI:** 10.3389/fpls.2020.00301

**Published:** 2020-03-24

**Authors:** Claudia Renate Stange, Manuel Rodriguez-Concepcion, Li Li

**Affiliations:** ^1^Department of Biology, University of Chile, Santiago, Chile; ^2^Centre for Research in Agricultural Genomics, Barcelona, Spain; ^3^Plant Breeding and Genetics Section, School of Integrative Plant Science, Cornell University, Ithaca, NY, United States

**Keywords:** plastid differentiation, carotenoid synthesis and degradation, metabolic flux, light, environmental cues

Because of the huge importance of carotenoids in plants and for human health, carotenoid metabolism and regulation have been studied extensively during the last 40 years. Carotenoids are produced and stored in plastids. Their contents are influenced not only by biosynthesis and degradation rates but also by the storage capacity of the plastid (which depends on the plastid type) and external cues such as light (which regulates the expression of biosynthetic genes and modulates plastid differentiation). Carotenoid accumulation also impacts plastid identity, and some of their degradation products are biologically active molecules that modulate many plant developmental processes. This Research Topic focuses on recent research done in these areas.

A review article summarizes current knowledge on transcriptional regulation of carotenoid biosynthesis including nearly all regulators identified to date in photosynthetic tissues, fruits, seeds, flowers, and storage roots. Future research perspectives emphasize on broadening the diversity of “model” systems for carotenoid research and integrating multi-omics data to identify additional carotenogenic transcriptional regulators and to map regulatory networks (Stanley and Yuan). These strategies, together with testing the function of known carotenogenic regulators discovered from one species in other distantly related species, will provide insights to fill the gaps on the understanding of transcriptional regulation of carotenoid biosynthesis in plants.

Contributors of this Research Topic also show that light deprivation in *Citrus* species accelerates peel degreening, chlorophyll degradation while reducing carotenoid contents (Lado et al.). Fruit shading downregulates the expression of key carotenogenic genes, but levels of transcripts for enzymes responsible to cleave carotenoids for the formation of β-citraurin or to supply carotenoid precursors via the methylerythritol 4-phosphate (MEP) pathway were not substantially affected by light deprivation. Their findings suggest that light stimulates carotenoid biosynthesis in the peel of citrus fruit, but that a light-independent regulation may also operate.

The Orange (OR) protein as a DnaJE1 family chaperone protein exerts multiple functions in governing carotenoid accumulation. OR regulates carotenoid biosynthesis by posttranslationally regulating phytoene synthase (PSY, the first and main rate-determining enzyme of the pathway). Moreover, OR promotes stable storage by inducing the formation of chromoplasts and preventing carotenoid degradation. In this Research Topic, a review article describes our current understanding of the molecular and biochemical bases underlying OR-induced carotenoid accumulation and the potential of modifying the gene and chromoplast sink toward the development of healthy food for a growing population (Osorio). Additionally, a mini-review emphasizes the role of the carotenogenic metabolic flux, its association with the “golden SNP” of melon CmOR, and its arrest during the process of melon fruit ripening to illuminate chromoplast differentiation and carotenoid accumulation (Feder et al.). Investigation of three differently spliced transcripts of cauliflower BoOR suggests the requirement of OR dimerization for OR protein stability in *planta*, the need of simultaneous presence of PSY interaction-domains for the holdase function of OR, and the possible formation of various BoOR heterodimers for an enhanced activity on carotenoid accumulation (Welsch et al.). Besides OR, other chaperones and the Clp protease complex are known to influence PSY proteostasis and carotenoid accumulation. In particular, both OR and Hsp70 chaperones impact the accumulation of carotenoids in transgenic tomato ripe fruit. However, the resulting carotenoid profile and chromoplast ultrastructure are different, suggesting that different chaperone families target different processes related to carotenoid metabolism and accumulation during tomato fruit ripening (D'Andrea and Rodriguez-Concepcion).

Carotenoids are required for chloroplast biogenesis. A study proposes that the MEP-independent mevalonate (MVA) pathway, which is not localized in plastids but in the cytosol, might provide GGPP isoprenoid intermediate which is then transported into plastids for carotenoids and chloroplast biogenesis in Arabidopsis embryos (Vranová et al.). Besides the well-known carotenoid-derived phytohormones (i.e., ABA and strigolactones) and signaling molecules (e.g., beta-cyclocitral), novel apocarotenoids are being identified to function as biologically-active regulators. A review paper describes the biosynthesis and biological functions of two of these regulatory apocarotenoids, and provides insights on their role in plant growth, development, and stress responses (Felemban et al.).

Future novel research focused on plastid differentiation and light-regulated processes will help further deciphering carotenoid biosynthesis and carotenogenic metabolic flux during plant growth and development.

In summary, this Research Topic provides valuable clues to advance in our understanding of the multi-level regulation of carotenoid biosynthesis, storage, and degradation by endogenous and environmental cues.

## Author Contributions

CS, LL, and MR-C wrote the manuscript.

### Conflict of Interest

The authors declare that the research was conducted in the absence of any commercial or financial relationships that could be construed as a potential conflict of interest.

